# Evaluation of Botox treatment in patients with chronic scrotal pain: Protocol for a randomized double‐blinded control trial

**DOI:** 10.1002/bco2.349

**Published:** 2024-04-24

**Authors:** Nicolai Skov Schiellerup, Hanne Kobberø, Karin Andersen, Charlotte Aaberg Poulsen, Mads Hvid Poulsen

**Affiliations:** ^1^ Department of Urology Odense University Hospital Odense Denmark

**Keywords:** Botox, BTX, chronical scrotal pain, CSP, minimal invasive procedure

## Abstract

**Background:**

Chronic scrotal pain is a common condition with a prevalence of 2.5–4.8% in male outpatients. Up to 40% of these patients report depressive symptoms and many feel isolated. Minimal invasive treatment is lacking, while spermatic cord injections of Botox® (BTX) have been proposed to offer long‐term pain relief.

**Study Design:**

This research protocol comprises a prospective multicentre, randomized, double‐blinded clinical trial drawing patients from other urological departments in the region of Southern Denmark.

**End Points:**

The primary end point will be reduction in pain evaluated by visual analogue score for pain at 3 months. Secondary end point will be length of effect of BTX injections along with changes in quality of life.

**Patients and Methods:**

The study will include 50 patients for randomization to either spermatic cord block with 100 IE BTX or sterile saline. All patients will prior to randomization undergo physical examination and will be asked to fulfil multiple questionnaires regarding pain and impact in daily life, that is, (1) visual analogue score for pain, (2) quality of life (EQ‐5D‐5L), (3) Chronic Prostatitis Symptom Index (NIH‐CPSI), (4) ICD‐10 depression questionnaire (MDI), (5) Likert global assessment scale, and (6) International Index of Erectile Function questionnaire. Physical examination and fulfilment of the questionnaires will be repeated multiple times throughout the study period of 12 weeks. After this time point, patients will be unblinded, and the control arm will be given the opportunity of cross‐over.

## 1 BACKGROUND

Chronic scrotal pain (CSP) is a common condition with a prevalence of 2.5–4.8%.^1^ CSP covers a variety of problems causing discomfort or pain in the scrotum originating from the testis or other parts of the scrotum including ductus deferens and epididymis. CSP is defined as intermittent or constant unilateral or bilateral scrotal pain lasting for more than 3 months, with a significant impact on the patients' daily life.^2^ Up to 40% of these patients report depressive symptoms and many feel isolated.^3^, ^4^ The aetiology of CSP is not fully understood but can be related to surgical procedures such as vasectomy, inguinal hernia repair, scrotal surgery, and abdominal and groin surgery, even though no apparent cause can be identified in 25–50% of the cases.^5^, ^6^

When no obvious source of pain is found, the most common approach usually begins with conservative treatment including analgesia, antibiotics, and in some cases, anticonvulsants and antidepressants.^5^, ^6^, ^7^, ^8^ When conservative treatment fails, a minimal invasive procedure with local anaesthesia (LA) cord block is often used before more invasive treatments such as denervation, vasovasostomy, orchiectomy, or epididymectomy are considered.^8^ Spermatic cord block with LA has proven to significantly reduce pain in patients with CSP.^9^ However, the effect of an LA cord block has limited duration, and it has been proposed that a longer lasting effect could be obtained with the use of OnabotulinumtoxinA (BTX), because BTX is widely used in pain management in diseases such as chronic pain syndrome, myofascial syndrome, headaches, arthritis, and neuropathic pain.^10^, ^11^, ^12^, ^13^, ^14^, ^15^

BTX interferes with neural transmission in two ways: (i) a direct way by blocking the release of acetylcholine, the principal neurotransmitter at the neuromuscular junction, causing muscle paralysis. Function can be recovered by the sprouting of nerve terminals and formation of new synaptic contacts which usually takes 2 to 3 months.^16^, ^17^ (ii) An indirect way by preventing the release of neurotransmitters other than acetylcholine (e.g., substance P, somatostatin, and serotonin) which is involved in the sensitization and stimulation of peripheral nociceptors which would otherwise stimulate the central nervous system and cause the sensation of pain.^10^, ^18^

The experience with BTX in patients with CSP is still limited and is not an integrated part of the standard care. An open‐label study has been conducted by Khambati et al.,^19^ where BTX was injected into the spermatic cord in 18 CSP patients. They found a significant reduction in pain measured by visual analogue score for pain at follow‐up after 1 and 3 months. However, pain and tenderness returned to baseline after 6 months.^19^ Following this, they conducted a randomized study comparing LA cord block with LA + BTX cord block in 64 patients and did not find a significant difference between pain reductions in the two arms.^20^ Though the overall difference in pain reduction was not significant, differences in the effect and duration of the treatment between the patients were described in both studies. The differences need to be further investigated, and it is not known if specific subgroups of CSP patients, based on the aetiology of the pain, have different effects of BTX treatments. In addition to this, the effect of BTX alone has not been described for CSP patients in a randomized manner but only tested in combination with LA versus LA alone. The clinical potential and use of BTX injections in CSP patients is therefore not fully described and BTX is not used on a regularly basis in the standard care of CSP patients.

### 1.1 Study design

**FIGURE 1:**
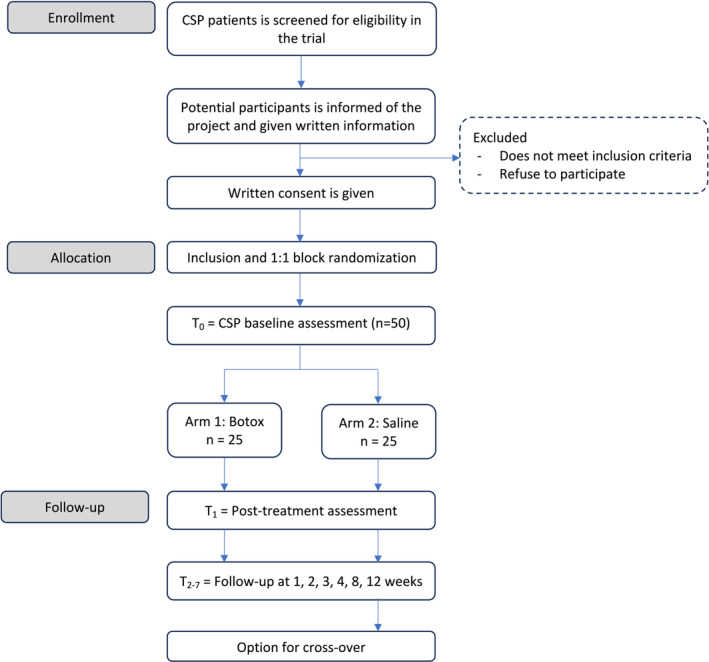
Flow of participants. CSP, chronic scrotal pain.

This study is a randomized, placebo‐controlled, double‐blinded trial covering the Region of Southern Denmark. Patients diagnosed with CSP experiencing successful spermatic LA cord block can be included. See flow of participants in Figure 1.

The aim of this study is to fully investigate the effect of chemical denervation with BTX injections on CSP and elucidate whether the effect is different between CSP patients based on the aetiology of the pain. By performing a randomized study comparing BTX injections with placebo (saline) injections in CSP patients, the effect of BTX alone will be investigated for the CSP patient group and subgroup analysis will elucidate differences between patients.

### 1.2 End points

Primary outcome is the success rate of BTX. According to the literature, an improvement of one point or more in pain, determined by VAS, when compared with baseline values will be considered a success.^19^, ^20^

Secondary outcomes are as follows:

- Duration of the effect; and
- Improvement of quality of life based on questionnaires (EQ‐5D‐5L, NIH‐CPSI, MDI, L‐GAS, and IIEF).

### 1.3 Eligibility criteria

Male subjects will be included in the study if he meets all the following inclusion criteria:

1. Age of 18–70 years;
2. Unilateral or bilateral scrotal pain >3 months;
3. No other identifiable pathology or cause of pain;
4. Insufficient effect of conservative treatment with unreduced pain measured by VAS; and
5. A positive effect of LA spermatic cord block with a reduction in VAS of minimum 1 point.

If any of the following criteria are met, the subject must not be included in the study:

1. Other pathological conditions in the scrotum, for example, varicocele, spermatocele, and epididymitis;
2. Interest in trying to conceive with partner in the following 6 months;
3. Local infection near the proposed injection site;
4. History with an allergic reaction to BTX and/or human serum albumin;
5. History of motor neuron disease or haemostatic disorder;
6. Active urogenital cancer; and
7. Current use of Botox injections which would result in a total dose of Botox higher than 300 IE in the span of 3 months.

## 2 METHODS

### 2.1 Intervention

After referral, patients will undergo a comprehensive workup including a thorough medical and psychological history and physical examination (i.e., inspection and palpation of penis, testis, funiculus, groin including inguinal channel, abdomen, digital rectal examination with evaluation of the prostate and pelvic floor muscles, hips, and column). In addition, investigations of lower urinary tract symptoms (i.e., uroflowmetry and bladder diary, furthermore urodynamics and cystoscopy if indicated), blood work (i.e., creatinine, haematocrit, white blood cells count, and C‐reactive protein, approximately 12 mL), and Doppler ultrasonography of the scrotum by an experienced radiologist to rule to examine for structural pathology and urine analysis (i.e., urine culture and test for chlamydia and gonorrhoea) will be included.

In case other pathological conditions are excluded, conservative treatment will be initiated with paracetamol 1000 mg four times a day, ibuprofen 400 mg three times a day, or alfuzosin 2.5 mg three times a day.^21^ If no pain relief occurs within 30 days, patients are offered funicle cord block with 10 mL lidocaine 20 mg/mL. In case of a significant decrease in scrotal pain measured by VAS, patients will be offered inclusion in the study if they fulfil the study eligibility criteria and provide written informed consent.

The patients will be informed at the consultation and given written information of

- Presentation of the project;
- Patient information material including links to the pamphlets from The National Committees on Health Research Ethics: “Før du beslutter dig” and “Forsøgspersoners rettigheder i et sundhedsvidenskabeligt forskningsprojekt”;
- Contact information for the project leader; and
- Questionnaires 1–6 as listed below.

Patients can consent to inclusion in the study at first consultation, but in case patients need time to consider participating, they will be given a proper amount of time and then contacted by phone. If they accept inclusion in the project, they will receive an electronic consent form by safe email (eBooks).

When patients accept inclusion in this study, they also accept retrieval of relevant information from patient records by the study manager, the sponsor, and representatives of the sponsor.

Standard information on age, sex, country of origin, length of symptoms, comorbidity, prior surgery, and medication used in the last 3 months will be collected from patient records.

Prior to BTX cord block, patients will answer the following questionnaires:

1. Visual analogue score for pain (VAS);
2. Quality of life (EQ‐5D‐5L);
3. Chronic Prostatitis Symptom Index (NIH‐CPSI);
4. ICD‐10 depression questionnaire (MDI);
5. Likert global assessment scale (L‐GAS); and
6. International Index of Erectile Function questionnaire (IIEF).

For randomization, the research coordinator will place an information sheet containing the randomization in sealed envelopes (1:1 block randomization). At first treatment, the research coordinator will open a sealed envelope revealing the randomization and prepare the solution needed. The patient and physician will be blinded for the treatment.

In both groups, injection of either BTX or sterile saline will be given approximately 1–2 cm distal to the superficial inguinal ring near the spermatic cord using a gauge 23 needle. Before injecting the solution, the syringe is aspirated to exclude intravascular injection. The intervention arm will be given an injection of 50 IU BTX (Botox®, AbbVie A/S, Copenhagen) in a solution of 5 mL sterile saline both lateral and medial to the funicle for a total amount of 100 IU BTX mixed in 10 mL sterile saline. The control arm will receive two injections of a total of 10 mL sterile saline in the same manner. Both groups will be observed for 30 min after the injections. After 3 months at the end of the follow‐up, patients in the placebo arm will be offered BTX injections if wanted.

### 2.2 Sample size determination

Given that the true success rate for BTX, regarding the primary end point, is at least 0.70, the placebo success rate among controls is 0.20. We will need to study 23 experimental patients and 23 control patients to be able to reject the null hypothesis that the failure rates for experimental and control subjects are equal with probability (power) 0.9. The type‐I error probability associated with this test of this null hypothesis is 0.05. Considering potential dropouts, we will include 25 patients per group.

### 2.3 Methods of data collection

The questionnaires that were answered prior to BTX cord block will be sent by secure email to all included subjects after 1, 2, 3, 4, 8, and 12 weeks. Physical examination will be repeated after 4, 8, and 12 weeks. The data will be stored in an approved logged database at the Odense Patient data Explorative Network only accessible by professional staff involved in the project.

### 2.4 Data analysis

Univariate analysis and multivariate analysis will be performed: *T*‐tests will be used to compare means between groups and chi‐squared tests to compare dichotomous variables, and to adjust for unequal distribution of parameters at baseline, multivariate regression models, linear models in case of an interval scaled outcome, and logistic regression in case of a dichotomous outcome will be performed.

## 3 DISCUSSION

Patients with CSP is often severely affected by the condition and many experiences depressive symptoms. These patients often pose a challenge as no apparent cause can be identified in 25–50% of the cases^5^, ^6^ which causes frustrations for both patients and clinicals. One study found that the average CSP patient will have seen an average of 4.5 urologist for the condition and have undergone 7.2 diagnostic interventions.^22^ Consequently, a thorough workup is necessary.

No efficient minimal invasive treatment is available, and in severe cases, patients are offered invasive treatments such as surgical denervation, orchiectomy, or epididymectomy.^8^ Therefore, BTX has been proposed as a new minimal invasive treatment, because BTX is used in the treatment of other pain syndromes. So far, only a few studies have examined the effect of BTX on patients with CSP with varying results. A pilot open‐label study reported significant pain relief in 72% of cases after 3 months with a reduction in effect after 3 months.^19^ A later randomized, double‐blinded, controlled trial showed no effect of BTX on scrotal pain after 1 month.^20^ In contrast to these two studies, we have chosen to refrain from lidocaine cord block prior to BTX injections as we do not want the analgetic effect of lidocaine to bias the effect of BTX.

Regular evaluation by questionnaires and physical examination after 4, 8, and 12 weeks has been chosen as BTX has shown to weaken muscles within 2 weeks^11^ and reduce neuropathic pain after 4 and 8 weeks.^23^ Hence, a close follow‐up seems appropriate.

The use of BTX as spermatic cord block is easy and minimally invasive and could offer patients long‐lasting pain relief. Long‐term treatment has shown efficacy and safety in patients with interstitial cystitis/bladder pain syndrome, which allows for retreatment if pain relief has been achieved.^24^ Only minor complications rated as CD‐1 by Clavien‐Dindo Classification^25^ such as local haematoma or infection are expected.

In conclusion, CSP has great bio‐psycho‐social consequences for many patients. Its complexity demands a thorough and standardized workup and treatment. We want to focus on the right treatment for the right patient by emphasizing the importance of a systematic approach when treating these patients. With this study, we want to show that BTX spermatic cord block might be an effective minimal treatment option for patients with CSP.

## 4 PARTICIPANTS DISCONTINUATION AND WITHDRAWAL CRITERIA

All patients are free to discontinue their study participation at any time during the study for whatever reason without their decision affecting their future treatment and care. If possible, the reason for withdrawal should be reported to the study coordinator.

The investigator can exclude a patient from the study after inclusion based on the following:

- Safety reasons (e.g., serious adverse event or another intercurrent illness); and
- Patient not willing to cooperate/non‐compliance.

The discontinuation should be documented and reported to the study coordinator. If a patient discontinues in the study, the subject number cannot be reused.

## 5 ETHICAL CONSIDERATIONS

The study will be conducted in compliance with the Declaration of Helsinki, Good Clinical Practice, and national laws and guidelines. The protocol was approved by The Regional Committees on Health Research Ethics for Southern Denmark before study initiation, and informed consent will be signed by all patients after thorough information has been given.

The use of BTX in pain treatment of CSP patients has been tested in small patient populations showing the treatment to be safe and not associated with severe side effects. The idea of using BTX in pain treatment is not new but well tested and documented for other medical conditions associated with other pain syndromes including bladder pain.^10^, ^11^, ^12^, ^13^, ^14^, ^15^ The value of BTX cord block is unsure, but BTX is widely used. Possible benefits of BTX are a significant reduction in pain evaluated by VAS and a more normal life.

Severe side effects observed when using BTX injections for other medical conditions are in general rare and treatable. When testing BTX injections in the two CSP studies, the complications observed, such as bleeding and local infections, can be considered minor, temporary, and treatable. Based on these data, we believe that it is responsible and safe to carry out the present study. We will only include patients that are thoroughly examined medically, patients where other medical reasons for the pain have been ruled out, and where the standard conservative treatment has shown no effect.

Patients will be informed about potential side effects, and every complication observed in the present study will be treated and followed closely. In addition, all complications will be registered in our database and reported.

The potential effect of our study is to improve treatment of pain in patients with CSP. Patients enrolled in the study will undergo both testing and minimal invasive surgery. This will be both time‐consuming and painful for the patients. However, at this point, the patient has been in pain for several months and there are no more conventional treatment options left, other than invasive surgery. In view of the potential benefits, the risk of minor complications such as infection and bleeding are to us acceptable.

We expect to observe a long‐lasting pain relief for up to 3 months in at least 70% of the patients in the intervention arm and in 20% of the patients in the placebo arm. We expect this to be sufficient to demonstrate the benefits of BTX injection for this group of patients. In addition, it will provide us with information about which patient group has the best effect of the treatment.

### 5.1 Safety

Based on former studies, BTX cord block has proven to be a safe minimal invasive procedure.^19^, ^20^ The studies found that the risk of complications is low. Unilateral transient hyperalgesia was reported, but no complications like bleeding or infection were registered. Events of adverse effects will be registered and treated until full resolution. Safety will be prospectively assessed and classified by Clavien‐Dindo Classification.^25^

An interim analysis will be conducted at 50% enrolment (*N* = 24 after loss to follow‐up, 12 patients per arm) accrual, and the decision to stop early will be governed by a significance level of 0.003. The decision to stop early will be guided both by interim results and clinical judgement, especially in the context of emerging, relevant literature.

Patients are covered by the Danish Patient Compensation.

## 6 DATA PROTECTION

Patient data are treated confidentially and will be stored according to the General Data Protection Regulation. No patient‐related information will appear in the analysis or published results. Signed consent forms and related paperwork will be uploaded in the database and saved in a locked cabinet. We expect to start inclusion in August 2024, and data will be deleted 10 years after this.

During the study, data from the patient's medical file will be registered in the database. Data include name, date of birth, CPR number, medical history related to the patients' CSP (date of first symptoms, symptoms, location of the pain, cause of the pain if known, results from physical examinations, description of scans of the scrotum, results from laboratory tests on blood and urine, and results from other medical tests), treatment (dates for treatment initiation and termination, medication given and dose, and effect of the individual treatments), results from questionnaires regarding pain and life quality, and effects and side effects related to the BTX or saline injection.

### 6.1 Publications policies

It is the intention to report our research findings on a continuous basis in relevant national and international medical journals and present our data at appropriate conferences. Rules for authorship are as stated in “Uniform requirements for manuscripts submitted to biomedical journals” (http://www.icmje.org). In addition, the study is registered at http://clinicaltrials.gov/ with identifier NCT05112081 in accordance with guidelines.

Co‐authorships will follow the Vancouver Guidelines ICMJE | Recommendations | Defining the Role of Authors and Contributors.

The results will be analysed and published as soon as possible after sufficient inclusion and the 12 weeks follow‐up period. Any result (positive, inconclusive, or negative) will be published. Individual clinicians may not publish data concerning their patients that are directly relevant to questions posed by the study until the Trial Management Group has published its report. Studies using the present study data to evaluate end points other than those specified in this protocol can be published by participating physicians after approval from the Trial Management Group.

## AUTHOR CONTRIBUTIONS

The study is initiated by N. S. S., K. A., and H. K. N. S. S. conceived and designed the study as well as drafted the manuscript. H. K., K. A., and M. H. P. conceived and designed the study, provided critical revisions, and approved the final version of the manuscript. C. A. P. provided critical revisions and approved the final version of the manuscript.

## CONFLICT OF INTEREST STATEMENT

The authors have no conflict of interests related to this publication.

## Data Availability

The datasets used and/or analysed during the current study are available from the corresponding author on reasonable request.
